# Positively Charged Biodegradable Polymersomes with Structure Inherent Fluorescence as Artificial Organelles

**DOI:** 10.1021/acs.biomac.4c00143

**Published:** 2024-05-02

**Authors:** Roy A. J. F. Oerlemans, Shoupeng Cao, Jianhong Wang, Yudong Li, Yingtong Luo, Jingxin Shao, Loai K. E. A. Abdelmohsen, Jan C. M. van Hest

**Affiliations:** Bio-Organic Chemistry, Department of Biomedical Engineering and Chemical Engineering & Chemistry, Institute for Complex Molecular Systems (ICMS), Eindhoven University of Technology, P.O. Box 513, 5600 MB Eindhoven, The Netherlands

## Abstract

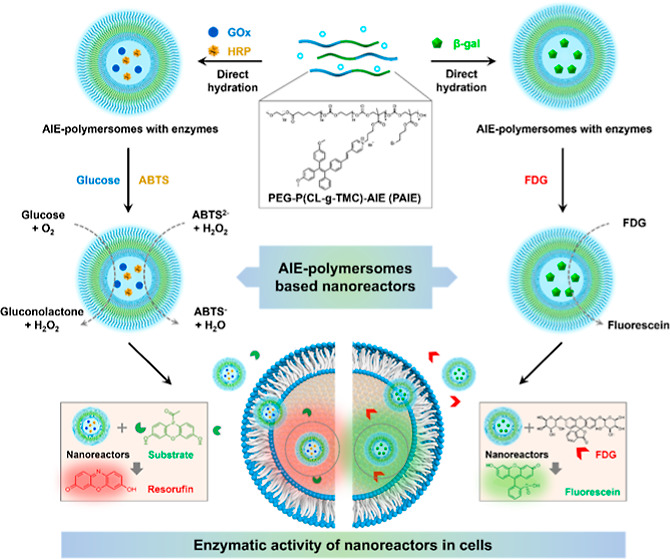

Polymersomes, nanosized polymeric vesicles, have attracted significant interest in the areas of artificial cells and nanomedicine. Given their size, their visualization via confocal microscopy techniques is often achieved through the physical incorporation of fluorescent dyes, which however present challenges due to potential leaching. A promising alternative is the incorporation of molecules with aggregation-induced emission (AIE) behavior that are capable of fluorescing exclusively in their assembled state. Here, we report on the use of AIE polymersomes as artificial organelles, which are capable of undertaking enzymatic reactions in vitro. The ability of our polymersome-based artificial organelles to provide additional functionality to living cells was evaluated by encapsulating catalytic enzymes such as a combination of glucose oxidase/horseradish peroxidase (GO*x*/HRP) or β-galactosidase (β-gal). Via the additional incorporation of a pyridinium functionality, not only the cellular uptake is improved at low concentrations but also our platform’s potential to specifically target mitochondria expands.

## Introduction

Polymersomes are a class of nanoparticles with great potential in artificial cell and biomedical research.^1−6^ Due to their nanoscale size, they are equipped with fluorescent dyes so that they can be visualized using standard confocal microscopy techniques. Examples of polymersomes and dyes used to label them are vast in the literature. One example from our lab is poly(ethylene glycol)-*block*-poly(caprolactone-gradient-trimethylene carbonate) (PEG-P(CL-*g*-TMC))-based polymersomes, which can be coassembled with block copolymers that are prefunctionalized with the BODIPY dye.^7^ However, the phenomenon of dye leaching poses challenges in attributing the fluorescent signal to either the particle itself or the dye/dye–polymer conjugate. A potential solution to this issue involves the utilization of aggregation-induced emission (AIE) molecules. AIE is characterized by fluorescence emission occurring exclusively in the assembled state, facilitating both visualization and confirmation of particle integrity.^8,9^ In contrast to aggregation-caused quenching, which leads to a decreased fluorescence of fluorophores when they are trapped in a solid-like state, AIE units need to be in this state to exhibit fluorescence. If not aggregated, AIEgens are not fluorescent since they contain rotatable bonds that prevent excitation. Upon aggregation, the freedom of movement is excluded, and excitation of such dyes results in a strong fluorescence. Another advantage of AIEgens is that they are commonly more photostable compared to regular dyes, allowing microscopy tracking over longer timeframes. Recently, AIE features have been included in polymersomes, with the initial studies published by Li et al.^10^ Subsequently, our group applied this technique to the biomedical context, by designing particles that exhibited both fluorescence and photodynamic therapy (PDT) capabilities, while being accompanied by an active motor function.^11^ A distinct subclass of AIE-modified PEG-P(CL-*g*-TMC)-based polymersomes was prepared. Specifically, the tetraphenylethylene (TPE) moiety as the hydrophobic segment was incorporated into the block copolymer PEG-P(CL-*g*-TMC), which displays AIE behavior.^12,13^ Following their cellular uptake, these particles exhibited exclusive localization within the mitochondria and improved PDT.^13,14^ Notably, PEG-P(CL-*g*-TMC)-based polymersomes demonstrate semipermeability, as indicated by previous data from our lab.^13,14^ This characteristic facilitated their use as nanoreactors or artificial organelles.

**Figure 1:**
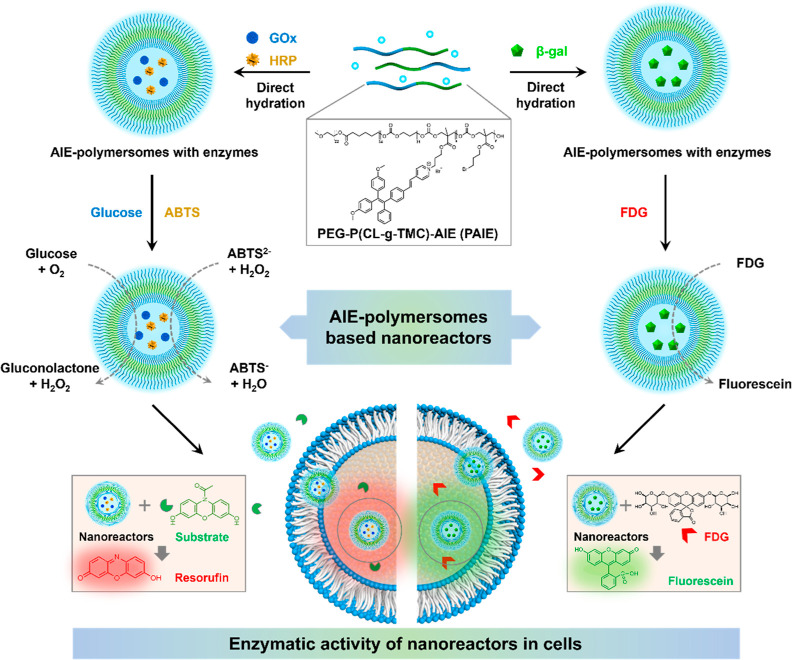
Schematic illustration of the design of AIE polymersomes as artificial organelles via enzyme encapsulation (GO*x*/HRP and β-gal) and their intracellular catalytic activity in the presence of glucose and ABTS or fluorescein di-β-d-galactopyranoside (FDG).

In this paper, we employ the platform of pyridinium functional-AIE-PEG-P(CL-*g*-TMC) polymersomes (AIE_*n*_ polymersomes) to create an artificial organelle with enzymatic functionality, capable of executing reactions within living cells (Figure 1). In our approach, each copolymer was labeled with an AIE and a pyridinium moiety, providing the polymersomes with a permanently positive charge. Consequently, the AIE polymersomes were readily taken up by living cells even at very low concentrations (as low as 50 μg/mL). An additional benefit of pyridinium inclusion involves the specific targeting of mitochondria. Pyridinium, as a polar lipophilic cation, enables molecules/nanocarriers to penetrate and accumulate selectively in the membrane of the mitochondria.^15−18^ To transform our AIE polymersomes into artificial organelles, we encapsulated enzymes in the hydrophilic lumen of the AIE polymersomes. As a model cascade, we employed the enzyme glucose oxidase/horseradish peroxidase (GO*x*/HRP). Furthermore, β-galactosidase (β-gal) was selected as well. The catalytic activity of AIE polymersomes in living cells was investigated thereafter. Owing to the positive charge, the polymersomes preferentially accumulated intracellularly, followed by generating fluorescent products in the presence of corresponding substrates. Through these investigations, we demonstrate the versatility of these polymersomes in imparting additional functionality to living cells.

## Experimental Section

### Materials

Poly(ethylene glycol)methyl ether (mPEG, *M*_n_ = 1 kDa) was acquired from Rapp Polymers. The dialysis membrane with a molecular weight cutoff (MWCO) of 12,000–14,000 Da was obtained from Spectra/Pro. Dulbecco’s modified Eagle’s medium (DMEM), phosphate-buffered saline (PBS, pH 7.4), Hoechst 33342, no-mycoplasma fetal bovine serum (FBS), trypsin–EDTA, penicillin–streptomycin, and live cell imaging solution were procured from Thermo Fisher. All other chemicals were provided by Merck. The chemicals used in this work were utilized without further purification unless stated otherwise. Ultrapure Milli-Q water (Millipore, 18.2 MΩ·cm) was employed in this study.

### Synthesis of AIE-Incorporated Block Copolymers

The synthesis of the AIE block copolymers was accomplished via a modular polymerization approach, as presented in the literature for similar macromolecules (Scheme S1). The PEG-P(CL-*g*-TMC) copolymer was first synthesized and used as the structural basis according to our previous report.^13,14^ Then, the PEG-P(CL-*g*-TMC) copolymer was utilized as a macroinitiator, copolymerized with bromide-functional TMC, and catalyzed by 1,8-diazabicyclo(5.4.0)undec-7-ene (DBU) at room temperature for 2 h, which generated reactive sites for AIE conjugation.^19^ To endow the block copolymer with AIE features, a pyridine-modified TPE derivative was first synthesized, following well-established literature procedures.^20^ Via a nucleophilic substitution reaction in DMF at 100 °C for 24 h, the bromides were replaced by quaternary pyridinium groups to provide the block copolymers with both AIE capacity and pyridinium-targeting moieties, yielding the final block copolymers (PEG-P(CL-*g*-TMC)-AIE_*n*_). Copolymer composition was calculated by using the protons of the AIE aromatic group (m, 9.40–9.01 ppm), (m, 8.12–7.95 ppm), (m, 7.70–7.50 ppm), (m, 7.41–7.32 ppm), (m, 7.16–6.90 ppm), (m, 6.69–6.60 ppm); AIE terminal ethoxy group (m, 3.77–3.73 ppm); macroinitiator (m, 4.97–4.79 ppm), (m, 4.28–4.02 ppm), (m, 2.53–2.39 ppm), (m, 2.38–2.27 ppm), (m, 2.08–1.98 ppm), (m, 1.73–1.61 ppm), (m, 1.45–1.34 ppm), (m, 1.31–1.14 ppm); PEG (m, 3.61–3.68 ppm); and terminal methyl unit (s, 3.38 ppm).

### Preparation of AIE Polymersomes via Direct Hydration

AIE polymersomes were prepared by direct hydration. A solution of PEG-P(CL-*g*-TMC)-AIE_*n*_ (PAIE_*n*_) in PEG-350 was prepared at a concentration of 10 wt % by weighing 40 mg of the copolymer into an Eppendorf tube along with 360 mg of PEG-350. To dissolve the block copolymers, the solution was mixed using a Gilson Microman E pipet at 50 °C. After complete dissolution and centrifugation, the copolymer solution (10 μL) was deposited at the bottom of a glass vial, followed by gentle stirring at a speed of approximately 250 rpm. Subsequently, a 100 mM NaCl (200 μL) solution was added to the solution. After mixing for 5 min, the polymersomes were formed at a polymer concentration of 5 mg/mL. To eliminate any large aggregates, the polymersomes underwent a 0.65 μm spin-filtration step. The AIE_*n*_ polymersomes were characterized by dynamic light scattering (DLS) and cryo-transmission electron microscopy (cryo-TEM).

### Preparation and Characterization of Enzyme-Loaded AIE Polymersomes

Enzyme-loaded polymersomes were prepared in a manner similar to that of the unloaded polymersomes. Specifically, a solution of PAIE_*n*_ was initially prepared in the presence of PEG-350 in an Eppendorf tube, followed by heating at 50 °C and mixing with a Gilson Microman E pipet. Subsequently, 10 μL of the copolymer solution was dispensed at the bottom of a glass vial, and the mixture was stirred gently (approximately 250 rpm). The enzymes (1 mg of GO*x* and 1.2 mg of HRP, 50 U/mL β-gal, respectively) to be encapsulated within the AIE polymersomes were dissolved in 100 mM NaCl (200 μL) and added to the copolymer solution. After being mixed for 5 min, the resulting enzyme-loaded AIE polymersomes were then purified to remove any free enzymes. Specifically, the enzyme-loaded polymersomes underwent purification via spin filtration utilizing a 300 kDa MWCO spin filter, followed by centrifugation at 3500 rcf at 4 °C. The polymersomes were then subjected to three washes with PBS and subsequently resuspended in PBS to achieve a final concentration of 4 mg/mL. The enzyme-loaded AIE polymersomes were characterized by cryo-TEM.

### Evaluation of the Catalytic Behavior of β-Gal-Loaded AIE Polymersomes

The catalytic activity of β-gal-encapsulated AIE polymersomes was evaluated in solution. Enzyme-loaded polymersomes (0.25 mg/mL polymersomes) were treated with different concentrations of the substrate (FDG, from 0.01 to 0.05 mg/mL). PBS-only and blank AIE polymersomes were used as controls. Fluorescence intensity at 510 nm was measured via a microplate reader-based assay.

### Evaluation of Catalytic Activity of GO*x*/HRP-Loaded AIE Polymersomes

The ABTS enzymatic assay, based on the formation of ABTS cation radicals, was used to determine the activity of GO*x*/HRP-loaded polymersomes.^21^ Briefly, 5 μL of a dispersion containing 2 mg/mL GO*x*/HRP-loaded polymersomes in PBS was mixed with either 20 μL of PBS or 20 μL of a 0.5 mg/mL trypsin solution from the bovine pancreas in PBS. Following this, the mixtures were incubated at 37 °C for 2 h. Subsequently, 24 μL of PBS, 50 μL of glucose (2 mM in PBS), and 1 μL of ABTS (10 mM in PBS) were added to each dispersion. The absorbance at 415 and 550 nm of the samples was monitored at 25 °C with intermittent shaking using a microplate reader.

### Cell Culture

Human cervical cancer cells (HeLa cells) were cultured in DMEM supplemented with 10% FBS and 1% penicillin–streptomycin (100 U/mL) in a Thermo Fisher cell incubator at 37 °C, 5% CO_2_, and 70% humidity. Prior to experimentation, cells were tested for mycoplasma, and no mycoplasma infections were detected.

### Intracellular Catalytic Activity

To further illustrate the capability of enzyme-loaded nanoreactors with live cancer cells, the β-gal-loaded polymersomes (0.05 mg/mL) alongside blank and controls (PBS, AIE polymersomes, and free enzyme) were incubated with HeLa cells (a typical cancer cell line as an example) for 3 h and washed with PBS three times. Subsequently, the substrate (FDG, 0.01 mg/mL) was added in the presence of a DMEM cell culture medium containing 10% FBS and 1% penicillin–streptomycin. The fluorescence of the resulting product was evaluated after washing and replacing the medium with a live cell imaging solution. To visualize the HeLa cells, the cell nuclei were stained with Hoechst 33342, followed by washing with live cell imaging solutions three times. The nanoreactor efficiency within cells and generation of fluorescent products were further qualitatively verified via confocal laser scanning microscopy (CLSM).

To assess the intracellular catalytic activity of GO*x*/HRP-loaded AIE polymersomes, HeLa cells were seeded and cultured in μ-slide 8 wells with 200 μL of the cell culture medium. The cells were then treated with a medium containing polymersomes (40 μg/mL) and incubated for 3 h. Then, a stock solution of Amplex Red at a concentration of 20 mM in DMSO was prepared. Following the incubation of the cells with the polymersomes and subsequent washing steps with PBS solution, 199 μL of the cell culture medium, along with 1 μL of substrate solution (Amplex Red), was added to the cells. The fluorescent product was characterized after washing steps using CLSM (Leica SP8X). Additionally, the cell nuclei were stained with Hoechst 33342 to facilitate the visualization of the HeLa cells.

## Results and Discussion

### Preparation and Characterization of AIE Polymersomes

Biodegradable AIE polymersomes, characterized by their inherent fluorescence, were assembled from PEG_22_-P(CL_34_-*g*-TMC_31_) copolymers functionalized with varying AIE segment lengths. These block copolymers were synthesized following a previously published procedure.^14^ The overall synthetic routes of the AIE block copolymers are illustrated in Figure S1. A pyridine-modified TPE derivative was synthesized. Concurrently, PEG-P(CL-*g*-TMC) copolymers were produced, using cationic ring-opening polymerization of caprolactone (CL) and trimethylene carbonate (TMC). Methoxy-poly(ethylene glycol)_22_ (mPEG_22_) served as the macroinitiator, and methanesulfonic acid (MSA) acted as a catalyst. The obtained PEG-P(CL-*g*-TMC) was then extended via the polymerization of a bromide-modified TMC derivative to introduce bromide reactive sites. This was followed by the conjugation of the pyridine-functionalized TPE derivative, resulting in the formation of a quaternized pyridinium moiety and imparting a permanent positive charge to the polymer (Figures S2–S6).

Previous studies have demonstrated that the morphology of the resulting assembly is significantly influenced by the degree of AIE-unit functionalization.^22^ For example, a block copolymer containing eight AIE units resulted in micellar structures, as the interpolymeric electrostatic repulsion likely leads to increased surface curvature and the consequent formation of micelles. In contrast, a block copolymer containing five AIE units formed vesicles. As the permeability could be affected by the thickness of the polymer membrane, we were interested in determining which minimal degree of polymerization of the AIE block would still lead to the formation of vesicles. As such, three copolymers were synthesized, comprising on average 2, 3, or 4 AIE units, denoted as AIE_2_-polymersomes, AIE_3_-polymersomes, and AIE_4_-polymersomes, respectively.

**Figure 2:**
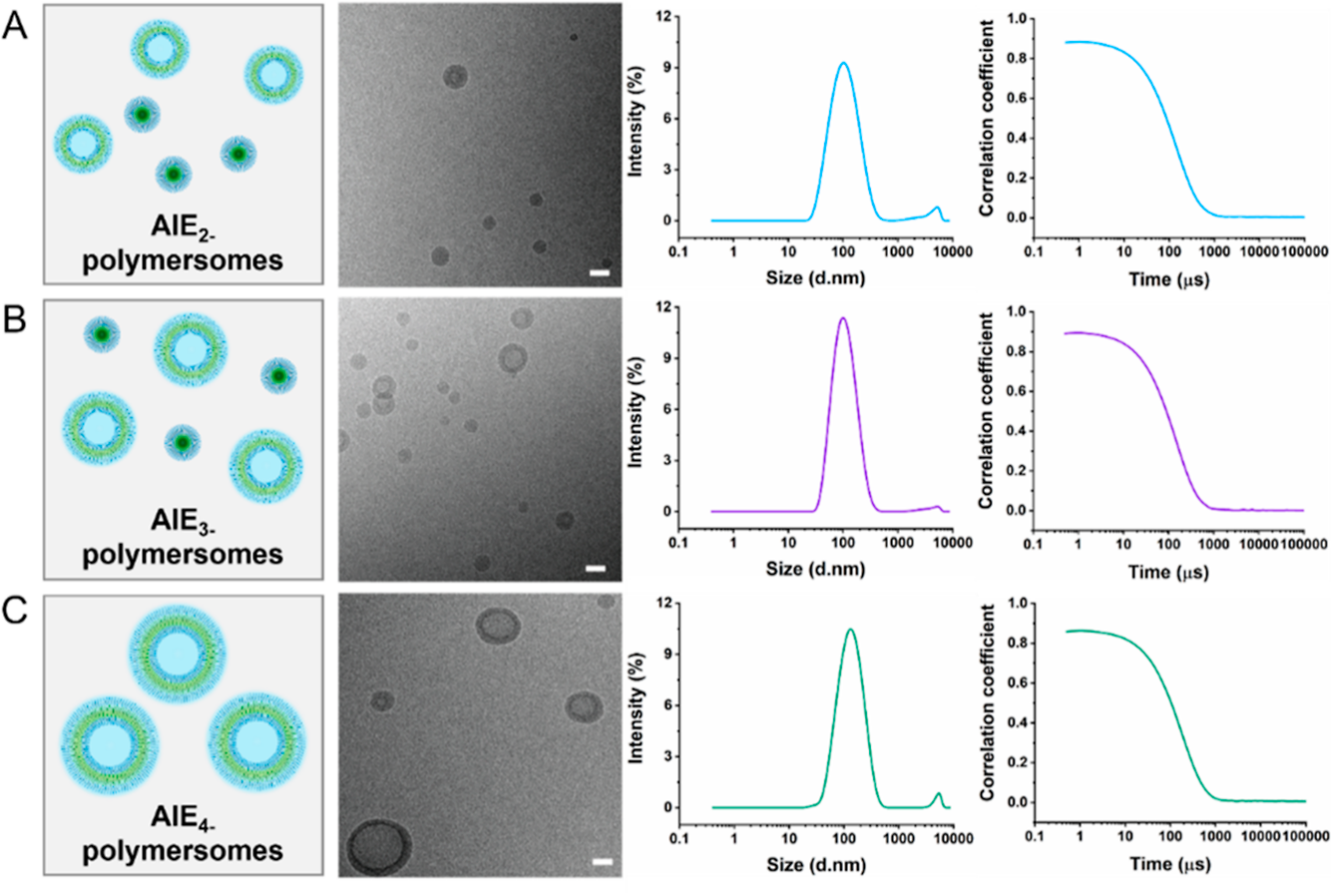
Characterization in the morphology and size of AIE_*n*_ polymersomes using cryo-TEM and DLS. (A) AIE_2_-polymersomes. (B) AIE_3_-polymersomes. (C) AIE_4_-polymersomes. Scale bar = 100 nm.

In general, the self-assembly process of PEG-P(CL-*g*-TMC) into polymersomes involves the direct hydration of a copolymer solution in oligo(ethylene glycol) (OEG) with an aqueous solution. Importantly, the self-assembly of AIE-functionalized copolymers into vesicles requires copolymer hydration with a saline solution, as hydration with Milli-Q water alone results in the formation of micelles, regardless of the number of AIE units incorporated. This can be explained by the ability of the salt to shield the positively charged pyridinium moiety through ionic interactions. In the absence of salt, increased electrostatic interactions/repulsions occur, leading to increased surface curvature and promoting the assembly of micellar structures rather than vesicular counterparts. We systematically evaluated the self-assembly process of each of the three distinct copolymers (PAIE_2_, PAIE_3_, and PAIE_4_) by directly hydrating them using a 100 mM NaCl solution. Subsequently, we analyzed the formed structures using cryo-TEM and DLS. As observed in Figure 2 and in Figure S7, the self-assembly of PAIE_2_ resulted in predominantly micellar structures and a small fraction of vesicles. The assembly of PAIE_3_ resulted in an increased fraction of vesicular structures and fewer micelles compared to PAIE_2_. Direct hydration of PAIE_4_ resulted in only vesicles, hereafter referred to as AIE polymersomes. The observations from the assembly of PAIE_2_ and PAIE_3_ can be explained by a slight imbalance between the hydrophobic and hydrophilic fractions, partially impairing the vesicular formation. AIE_4_-polymersomes were characterized by an average hydrodynamic size (*D*_h_) of approximately 125 nm (PDI = 0.28), whereas AIE_2_-polymersomes and AIE_3_-polymersomes were smaller in size (*D*_h_ = ca. 100 nm). This investigation demonstrated that the consequential morphological variations are influenced by the number of AIE units in the copolymers and emphasized the significance of fine-tuning the copolymers’ molecular structures to achieve the desired structures. Based on these findings, AIE_4_-polymersomes were used for the following experiments.

**Figure 3:**
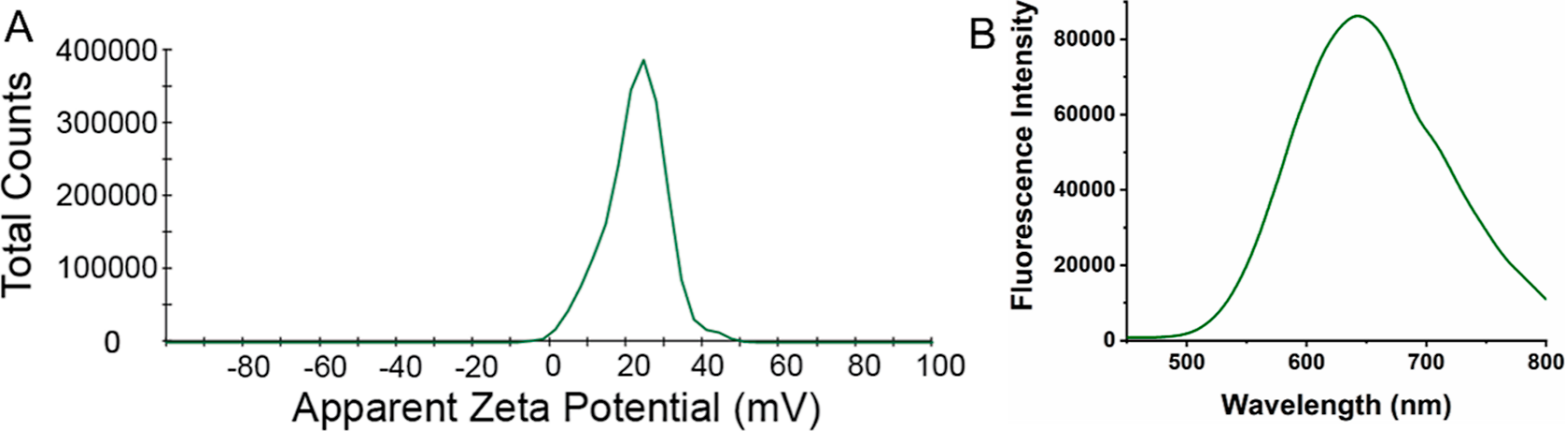
Characterization of AIE polymersomes. (A) Zeta-potential analysis. (B) Emission spectrum of AIE polymersomes (λ_Ex_ = 405 nm).

As anticipated, AIE polymersomes exhibited a net positive charge (ζ = 22.6 mV), as confirmed by zeta-potential analysis, owing to the presence of surface pyridinium moieties (Figure 3A). Subsequently, we verified the inherent fluorescence of our AIE polymersomes through a standard fluorescence emission scan (λ_Ex_ = 405 nm) (Figure 3B). The AIE polymersomes displayed inherent fluorescence and could be detected in the wavelength range from 600 to 700 nm, with a maximum emission wavelength of *ca*. 650 nm, which is suitable for cell imaging using CLSM.

**Figure 4:**
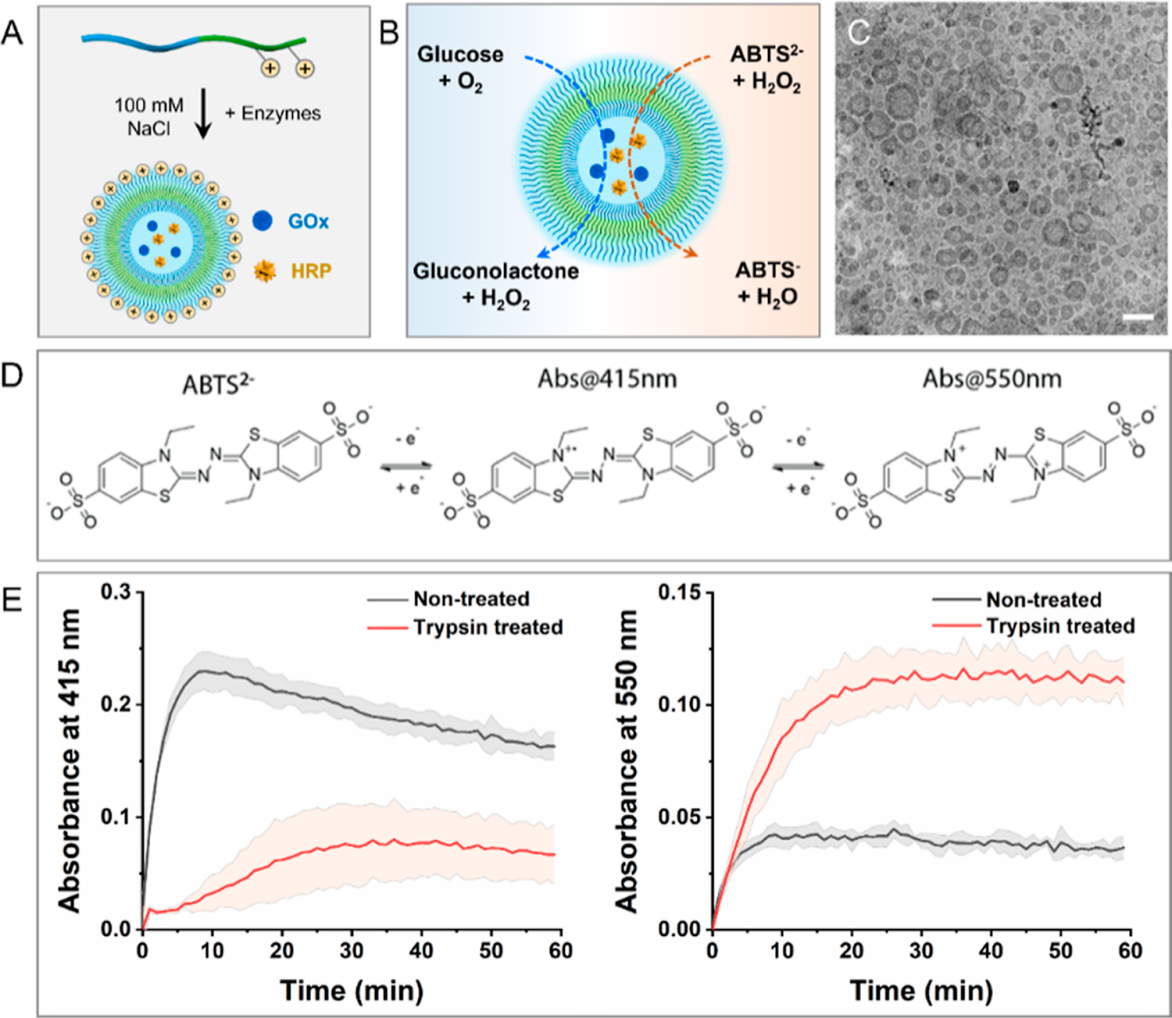
Enzyme encapsulation and enzyme cascade reaction of AIE polymersomes. (A) Schematic illustration of the enzyme encapsulation in positively charged AIE polymersomes. (B) Schematic presentation of the conversion of ABTS. (C) Cryo-TEM image showing GO*x*- and HRP-loaded AIE polymersomes. Scale bar = 100 nm. (D) Reaction scheme of the oxidation of ABTS. (E) Colorimetric readout of ABTS conversion determined at 415 nm (left) and 550 nm (right), respectively.

Having confirmed the physicochemical properties of AIE polymersomes and validated their inherent fluorescent features, we aimed to deploy them as nanoreactors by incorporating catalytic functionality. Two enzymes, GO*x* and HRP, were chosen for this purpose (Figure 4A). Due to their robust nature and the ability to assess their catalytic activity through colorimetric or fluorescent readouts, both enzymes are widely utilized in nanoreactor research.^14^ In a cascade fashion, in the presence of glucose and 2,2′-azino-bis(3-ethylbenzothiazoline-6-sulfonic acid) (ABTS), GO*x* catalyzes the oxidation of glucose into gluconolactone, producing hydrogen peroxide (H_2_O_2_). Subsequently, HRP utilizes generated H_2_O_2_ to oxidize ABTS, resulting in a fluorescent product (Figure 4B). To prepare the nanoreactors using the previously discussed direct hydration methodology, a 100 mM sodium chloride (NaCl) solution was employed with a total enzyme concentration of 11 mg/mL and a molar ratio of approximately 1:4 for GO*x* and HRP. Following self-assembly, a combination of spin filtration and protease treatment was employed to remove and deactivate nonencapsulated enzymes. While spin filtration is generally sufficient to remove nonencapsulated enzymes, the positively charged nature of our AIE polymersomes’ surface may increase the possibility of electrostatic-mediated adherence of enzymes. Therefore, protease treatment was necessary to deactivate surface-bound enzymes. Trypsin, as a protease, has been frequently used for the same reason in previous research.^23,24^ Specifically, a 3 h protease treatment was performed with an excess of trypsin. The integrity of the formed nanoreactors was confirmed using cryo-TEM, as shown in Figure 4C.

Next, glucose and ABTS solutions were added to the nanoreactors. Notably, ABTS can undergo two successive oxidations, single and double, resulting in readouts at two different wavelengths at 415 and 550 nm, for the single oxidized ABTS (ABTS^●–^) and doubly oxidized ABTS (ABTS^–^), respectively (Figure 4D). We set out to assess the catalytic activity of both trypsin-treated and nontreated nanoreactors (Figure 4E). Compared to the trypsin-treated nanoreactors, a substantial fluorescence signal was obtained with their nontreated counterparts at 415 nm. Hence, it can be inferred that not all enzymes were encapsulated by the AIE polymersomes; instead, some were found to be associated with the external surface of the membrane. Interestingly, this was not the case at 550 nm (indicative of double-oxidized product formation), as a higher fluorescent signal (and thus catalytic activity) was observed in the trypsin-treated nanoreactors compared to their nontreated counterparts. The discrepancy between the readouts at 415 and 550 nm is possibly due to the absence of a protein corona surrounding the trypsin-treated AIE polymersomes. This absence facilitates the diffusion of the substrate into the vesicle lumen, where the HRP concentration is comparatively higher. This elevated local concentration results in rapid double oxidation, leading to a more pronounced increase in absorbance at 550 nm in the trypsin-treated nanoreactors.

**Figure 5:**
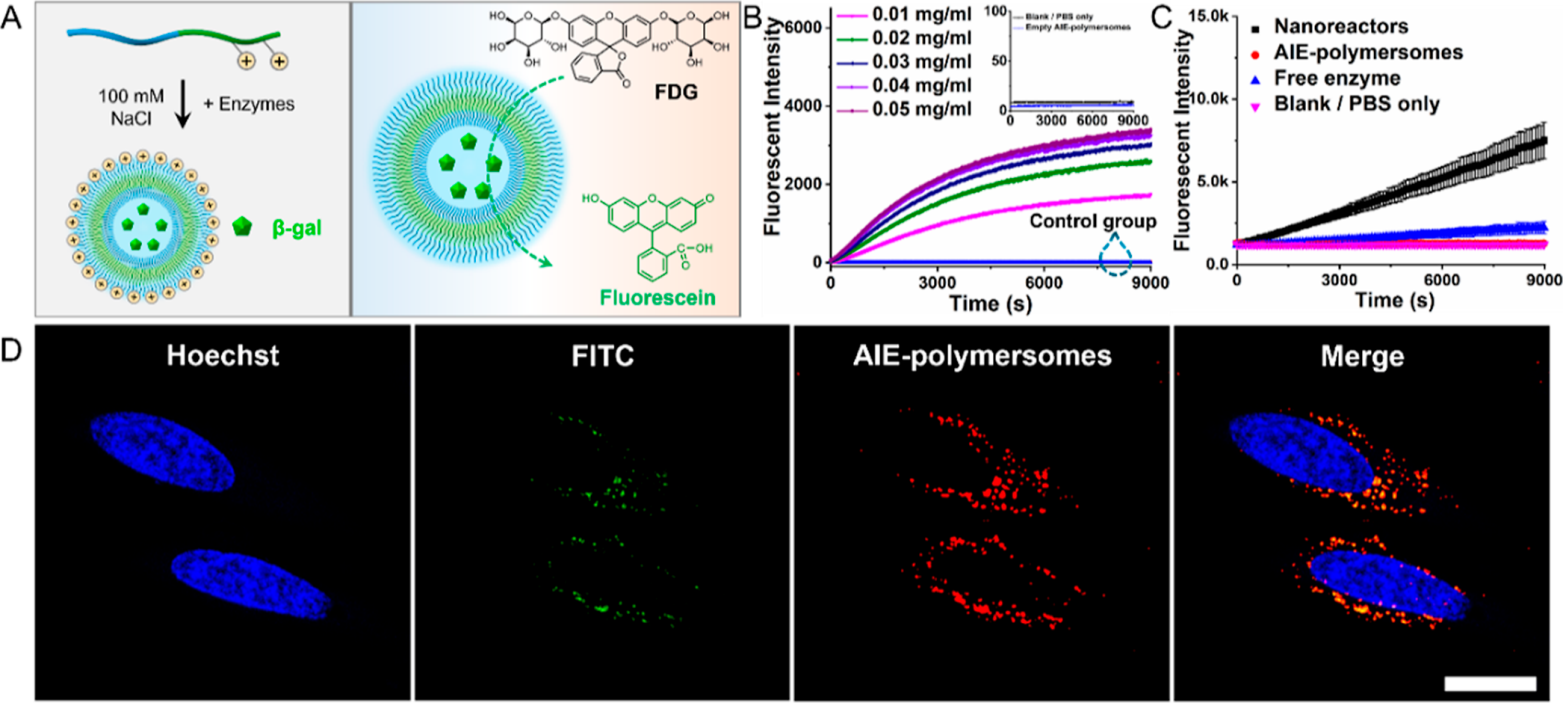
AIE polymersomes as nanoreactors through encapsulation of the enzyme β-gal. (A) Schematic illustration of β-gal-loaded AIE polymersomes converting the profluorescent substrate FDG to the fluorescent product. (B) Time course of fluorescence measured at 510 nm derived from β-gal-loaded AIE polymersomes with different concentrations of FDG and negative control groups (blank/PBS only and empty AIE polymersomes in zoom in figure, black, and blue lines). (C) Time course of fluorescence measured at 510 nm derived from HeLa cells incubated with β-gal-loaded AIE polymersomes (nanoreactors), empty AIE polymersomes, free enzyme, and PBS at 37 °C; red and purple lines overlap. (D) Evaluation of the enzymatic activity of β-gal-loaded AIE polymersomes inside HeLa cells as indicated by the occurrence of green fluorescence detected by CLSM (blue: Hoechst (nucleus); green: FITC; red: AIE polymersomes). Scale bar = 20 μm.

By integration of polymersome nanoreactors with living cells, they have the potential to be used for enzyme replacement therapy and correcting dysfunctional metabolic pathways. After the catalytic activity of AIE-polymersome-based nanoreactors was evaluated, the intracellular activity was examined in HeLa cells. The biocompatibility of AIE-polymersome-based nanoreactors with HeLa cells, as well as the cellular uptake and mitochondrial targeting, has already been demonstrated previously.^12^ Consequently, we directly tested the activity in HeLa cells. First, a single enzyme, namely, β-gal (50 U/mL), was loaded during the formation of AIE polymersomes by direct hydration in 100 mM NaCl. β-Gal is an enzyme, able to hydrolyze the pro-fluorescent substrate, FDG, to produce a fluorescent product, fluorescein (Figure 5A). To assess the activity of the nanoreactors, the profluorescent substrate FDG was used and the reaction was monitored using a microplate reader. Upon hydrolysis, fluorescent signals appeared, and the fluorescence intensity increased gradually in response to the substrate concentrations (ranging from 0.01 to 0.05 mg/mL), exhibiting a substrate concentration-dependent behavior (Figure 5B). The blank (without AIE polymersomes and PBS only) and empty AIE polymersomes (without enzyme encapsulation) were used as negative controls and were coincubated with the 0.05 mg/mL substrate. As expected, little to no increase in the fluorescence intensity was detected in the negative controls.

The capability of the β-gal-loaded nanoreactors to operate in living cells was then investigated. β-Gal-loaded polymersome-based nanoreactors were incubated with HeLa cells for 3 h and washed with a PBS solution, following the addition of profluorescent substrate FDG. Through quantitative analysis using a microplate reader, the fluorescence intensity in the presence of nanoreactors was 7.4 times higher compared to empty AIE polymersomes and the negative control (PBS) group, whereas the free enzyme only showed a 2.2-fold increase in fluorescence intensity (Figure 5C). Compared to the free enzyme, the positively charged AIE polymersomes are readily taken up by the HeLa cells, which can explain the higher fluorescence intensity generated in the group of nanoreactors. Subsequently, the nanoreactor efficiency within HeLa cells was further verified via CLSM imaging. To visualize the HeLa cells, the cell nucleus was stained by Hoechst. As shown in Figure 5D, red fluorescence originated from the inherent fluorescence of AIE polymersomes and green fluorescence was from the product through the catalytic reaction. HeLa cells containing the nanoreactors displayed obvious green fluorescence.

**Figure 6:**
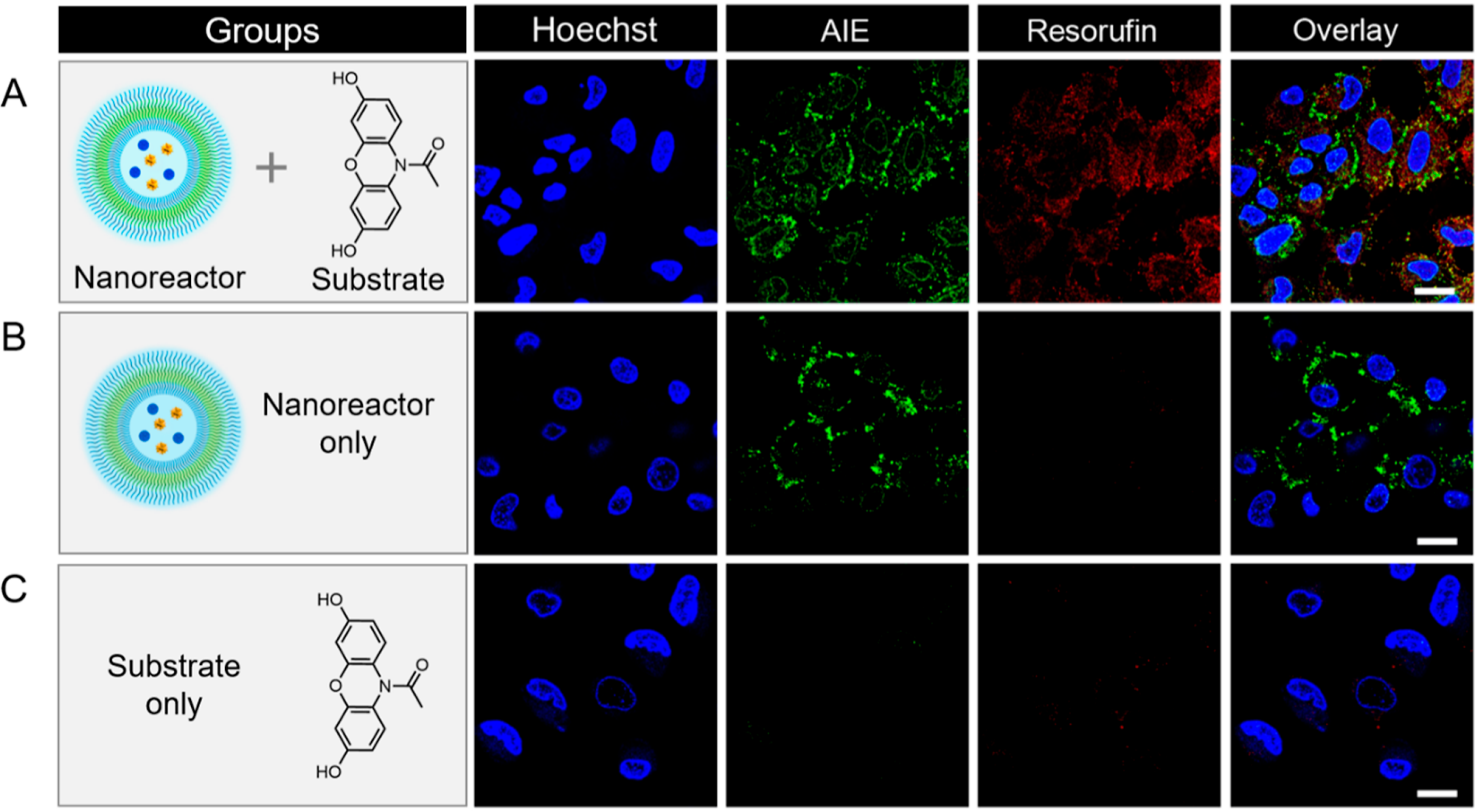
CLSM images of intracellular catalytic activity of AIE-polymersome-based nanoreactors. (A) HeLa cells treated with nanoreactors, followed by addition of the substrate. (B) Nanoreactors incubated with HeLa cells for 3 h in the absence of the substrate. (C) Only the substrate was added to the HeLa cells. Scale bar = 20 μm.

Finally, the GO*x*- and HRP-loaded AIE polymersomes (40 μg/mL) without pretreatment with trypsin were incubated with cells in a cell culture medium for 3 h. Subsequently, the cells were washed and treated with Amplex Red (100 μM) for 2 h. CLSM imaging indicated the formation of resorufin as shown by the increase in red intracellular fluorescence (Figure 6). When no substrate was added to the cells, the red fluorescence was not observed. Furthermore, substrate conversion was not observed in cells that had not previously been incubated with the catalytic AIE polymersomes.

## Conclusions

In summary, we have demonstrated the design of AIE polymersomes as artificial organelles. First, we evaluated the impact of the number of AIE units on the self-assembly behavior of PEG-P(CL-*g*-TMC) polymersomes and identified a copolymer composition for the exclusive formation of vesicles. Thereafter, we demonstrated the nanoreactor application of our AIE-polymersome platform by encapsulating enzymes, either a combination of GO*x* and HRP, or β-gal, and subsequently assessed their catalytic activity. Finally, we employed our AIE polymersomes as artificial organelles, demonstrating their uptake and ability to undertake reactions in living cells.

## Supporting Information Available

Instruments and characterizations, synthetic route of block copolymers, characterization of polymers, and cryo-TEM images (PDF)
