# Supplementary material for: Positively Charged Biodegradable Polymersomes with Structure Inherent Fluorescence as Artificial Organelles
Source: Biomacromolecules. 2024 May 2;25(5):3055–62. doi: 10.1021/acs.biomac.4c00143 (PMC11094732; doi:10.1021/acs.biomac.4c00143)
Supplement: Supplementary file 1 — bm4c00143_si_001.pdf [file bm4c00143_si_001.pdf]

Supporting Information

Positively Charged Biodegradable Polymersomes with Structure Inherent Fluorescence as Artificial Organelles

Roy A. J. F. Oerlemans‡, Shoupeng Cao‡, Jianhong Wang, Yudong Li, Yingtong Luo, Jingxin Shao, Loai K. E. A. Abdelmohsen*, Jan C. M. van Hest**

Bio-Organic Chemistry, Department of Biomedical Engineering and Chemical Engineering & Chemistry, Institute for Complex Molecular Systems (ICMS), Eindhoven University of Technology, P.O. Box 513, 5600 MB Eindhoven, The Netherlands

‡These authors contributed equally to this work

*Corresponding authors: j.shao@tue.nl, l.k.e.a.abdelmohsen@tue.nl, j.c.m.v.hest@tue.nl

Materials and Methods:

Proton Nuclear Magnetic Resonance Spectroscopy (^1H NMR): ^1H NMR spectra were conducted on a Bruker (400 MHz) spectrometer with a Bruker Sample Case auto-sampler, using TMS as the internal standard.

Dynamic Light Scattering (DLS): Zeta potential and size distribution of the polymersome samples were determined using a Zetasizer (model Nano ZSP, Malvern Instruments) equipped with a 633 nm He-Ne laser and an avalanche photodiode detector. Zetasizer software was employed for processing and analyzing the corresponding data.

Cryogenic Transmission Electron Microscopy (cryo-TEM): Morphological characterization of the polymersome samples was performed using the TU/e CryoTitan equipped with a field-emission gun operating at 300 kV, an autoloader station, and a post-column Gatan. For cryo-TEM sample preparation, 3 μL of the polymersome solution was pipetted onto the grid and blotted in a Vitrobot MARK IV (Thermo Fisher Scientific) at 100% humidity to remove the excess solution.

Ultraviolet-visible Spectroscopy (UV-vis spectra): UV-vis spectra were acquired using a UV-vis spectrophotometer (V-650, Jasco).

Microplate Reader: The catalytic activity of the polymersome samples was assessed using a microplate reader (Safire2, TECAN).

Confocal Laser Scanning Microscopy (CLSM): Fluorescent images of living cells were observed and recorded using a CLSM (Leica TCS SP8X).

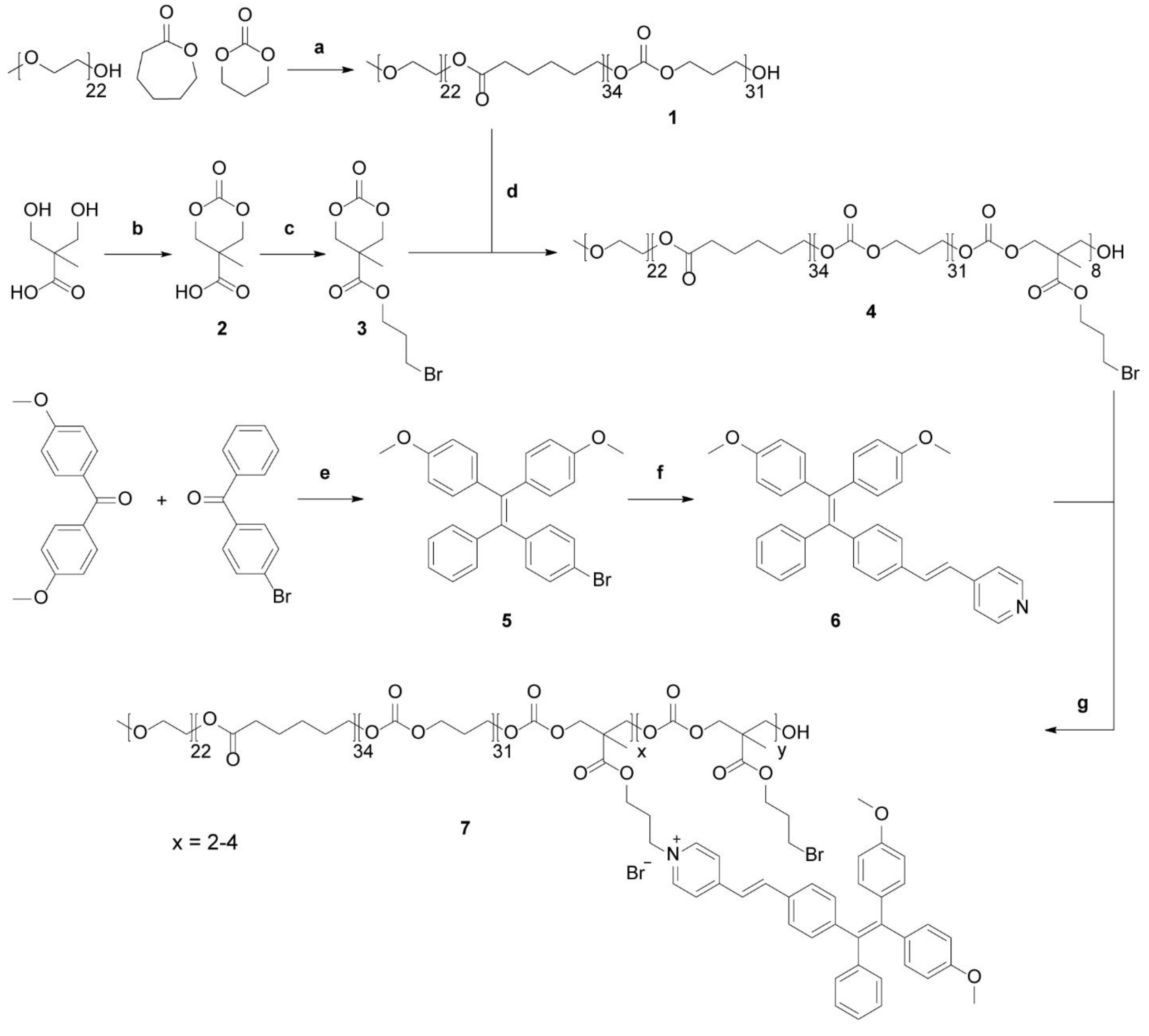

Figure S1. Synthetic route of AIE-functionalized PEG-P(CL-g-TMC) amphiphilic block copolymers ($PAIE_n$). a. MSA, CH_2Cl_2, room temperature, o/n, 75%. b. i. Et_3N, 1,1-carbonyldiimidazole, room temperature, 0.5 h; ii. AcOH, 60 ˚C, o/n, 67%. c. i. oxalyl chloride, DMF, THF, room temperature, 1 h; ii. 3-bromopropanol, 4-dimethylaminopyridine, Et_3N, 0 ˚C-room temperature, o/n, 84%. d. 1,8-diazabicyclo[5.4.0]undec-7-ene, CH_2Cl_2, room temperature, 2

h, 81%. e. Zn powder, $TiCl_4$, THF, -10 ˚C - 80 ˚C, o/n, 37%. f. $Pd(OAc)_2$, tri(o-tolyl)phosphine, 4-vinylpyridine, Et_3N, DMF, 100 ˚C, o/n, 91%. g. DMF, 100 ˚C, 24 h, 58%-73%.

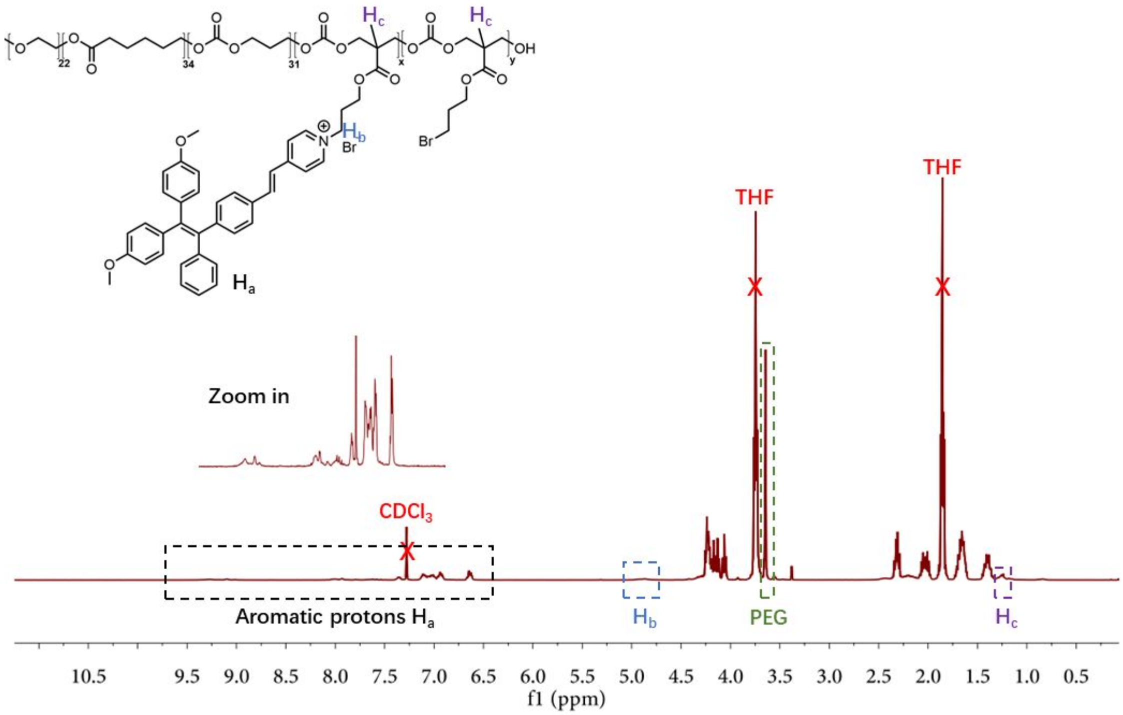

Figure S2. ^{1}H NMR spectrum of typical amphiphilic block copolymers (PAIE). The degree of quaternization was determined by the integral values of the protons in mPEG (3.57 ppm) and the protons in the AIE aromatic group (6.5 to 9.5 ppm). The quaternization efficiency was subsequently determined by comparing the integral values of the protons in the AIE aromatic group (6.5 to 9.5 ppm) with the total number of protons in the TMC-CH_3 group (1.21 to 1.31 ppm).

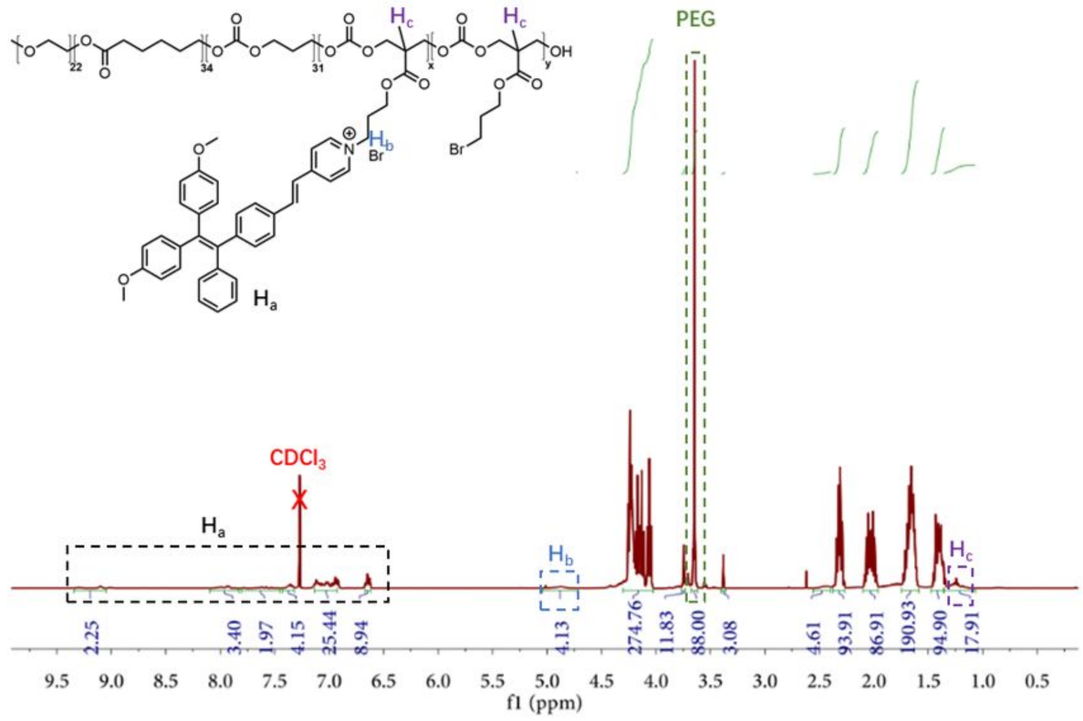

Figure S3. ^{1}H NMR spectrum of amphiphilic block copolymer PAIE$_2$.

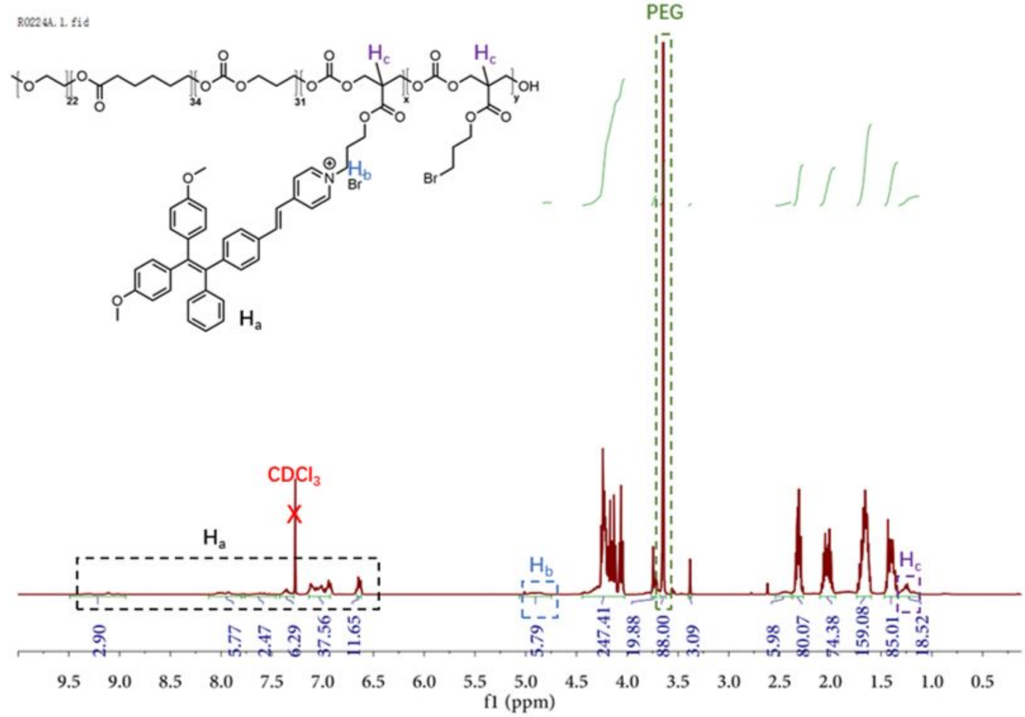

Figure S4. ^{1}H NMR spectrum of amphiphilic block copolymer PAIE$_3$.

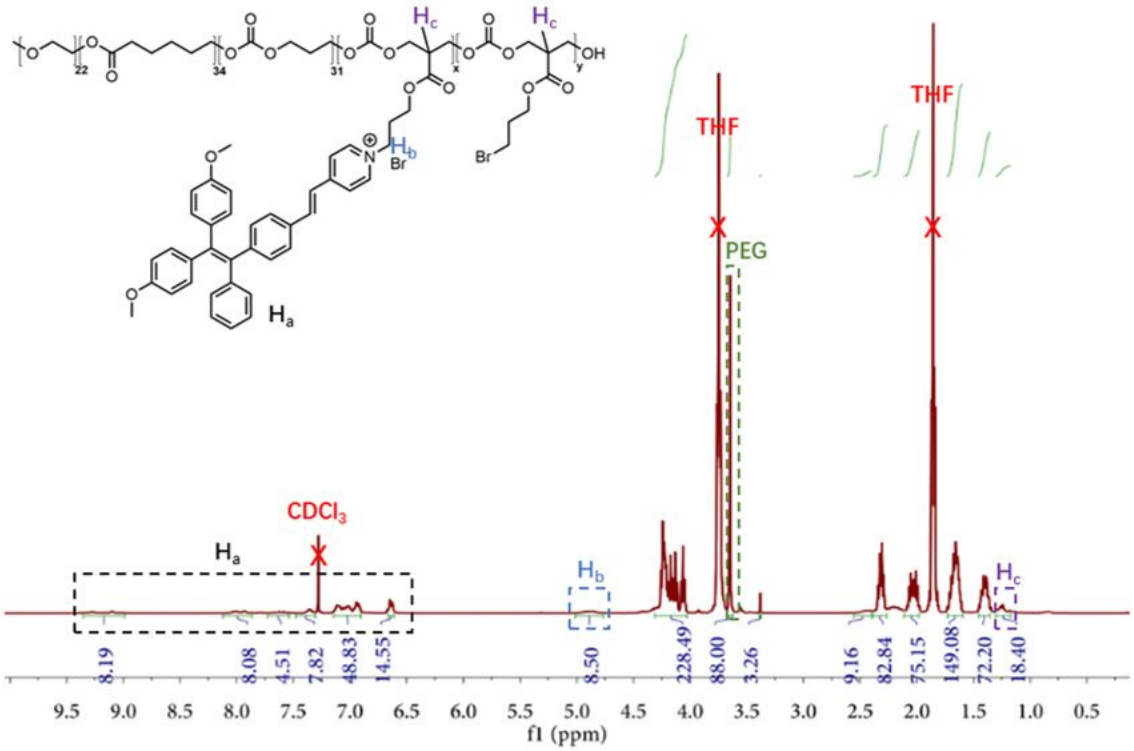

Figure S5. ^{1}H NMR spectrum of amphiphilic block copolymer $PAIE_4$.

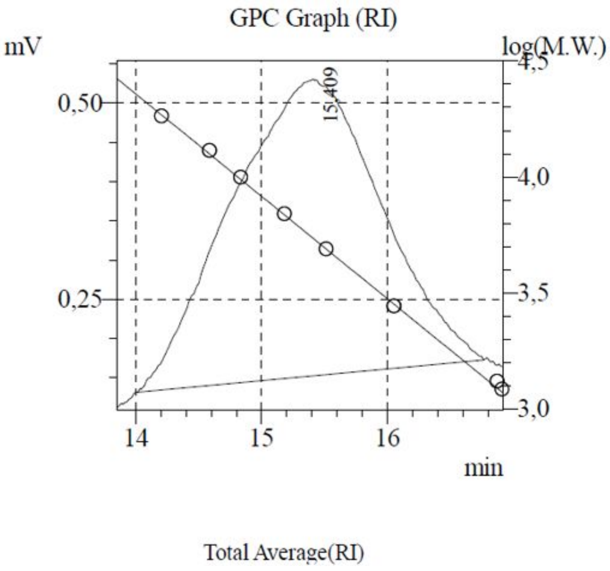

Figure S6. GPC characterization of amphiphilic block copolymer PAIE.

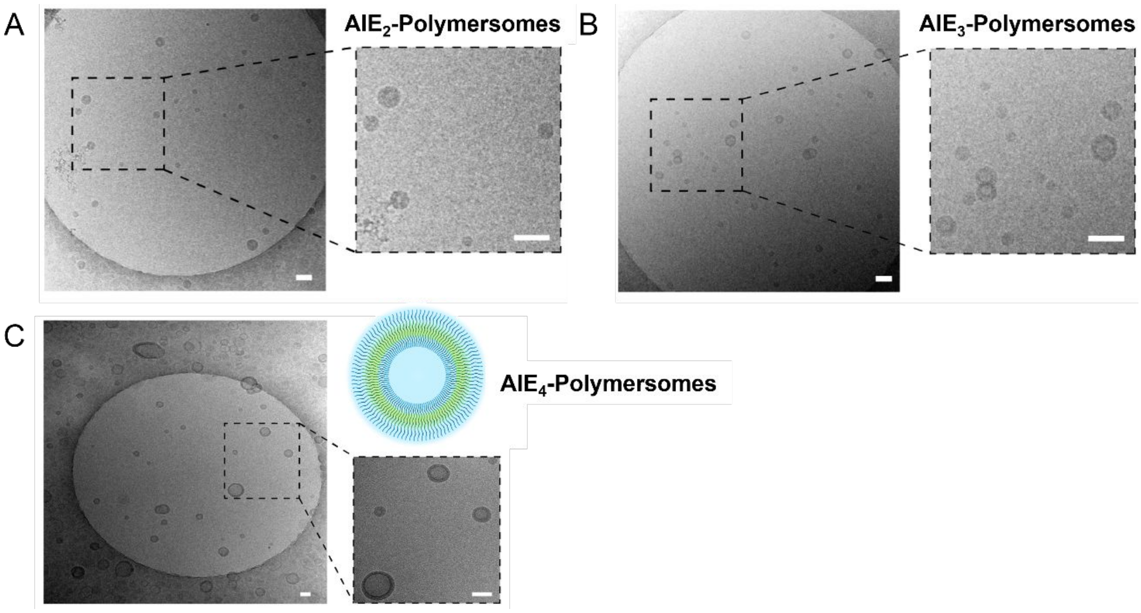

Figure S7. Cryo-TEM images of assemblies prepared by AIE_2-polymersomes (A), AIE_3-polymersomes (B), and AIE_4-polymersomes (C). Scale bar = 100 nm.
